# Incidental Finding of a PSMA-Positive Pancreatic Cancer in a Patient Suffering from a Metastasized PSMA-Positive Prostate Cancer

**DOI:** 10.3390/diagnostics11010129

**Published:** 2021-01-15

**Authors:** Simon Sirtl, Andrei Todica, Harun Ilhan, Michal Zorniak, Peter Bartenstein, Julia Mayerle

**Affiliations:** 1 Department of Medicine II, University Hospital, 81377 LMU Munich, Germany; michal.zorniak@med.uni-uenchen.de (M.Z.); julia.mayerle@med.uni-muenchen.de (J.M.); 2 Department of Nuclear Medicine, University Hospital, 81377 LMU Munich, Germany; andrei.todica@med.ui-muenchen.de (A.T.); harun.ilhan@med.uni-muenche.de (H.I.); peter.bartenstein@med.uni-muenchen.de (P.B.)

**Keywords:** PSMA, pancreatic cancer, PET

## Abstract

An 82-year-old man suffering from prostate cancer that was scheduled for a radioreceptor-ligand therapy (RLT) presented with jaundice to our service. An abdominal ultrasound (US) revealed obstructive extrahepatic cholestasis due to a solid lesion located in the uncinate process of the pancreas. The Prostate Specific Membrane Antigen (PSMA) PET/CT prior to RLT showed multilocular PSMA positive tumor lesions in the lymph nodes, the lung and the pancreas. On request of the cancer board, an Endoscopic Ultrasound (EUS)-guided Fine-Needle Aspiration (FNA) of the pancreatic mass was performed revealing invasive pancreatic ductal adenocarcinoma incompatible with a prostate cancer metastasis leading to the diagnosis of a PSMA positive pancreatic ductal adenocarcinoma.

An 82-year-old man suffering from prostate cancer was planned for Prostate Specific Membrane Antigen (PMSA) radioligand therapy. The 18F-PSMA PET/CT (Figure 1) scan revealed multilocular PSMA positive tumor lesions (maximum intensity projection; 1A) in the lung (1B), the mediastinal (1B) and the pancreas (1C) as well as in abdominal/iliac lymph nodes (1D). In addition, the patient presented with jaundice and the abdominal ultrasound (US) revealed obstructive extrahepatic cholestasis due to a solid lesion located in the uncinate process of the pancreas. The increased tracer uptake in the uncinate process suggested a prostate cancer metastasis as cause of obstructive jaundice. PSMA uptake in the uncinate process (SUVmax 15; SUVmax: Maximum Standard Uptake Value as a measurement for physiological quantification of radioactivity concentrations) equaled uptake in lymph nodes (SUVmax up to 17) and was even higher than the pulmonary metastasis (SUVmax 7). To exclude secondary malignancy in the pancreatic head, the prostate cancer multidisciplinary team asked for an Endoscopic Ultrasound (EUS) guided biopsy of the uncinate process. The cytoblock preparation showed an invasive pancreatic ductal adenocarcinoma incompatible with a prostate cancer metastasis leading to the diagnosis of a PSMA positive pancreatic ductal adenocarcinoma. PSMA is not only expressed in prostate cancer cells but also in the neovascular endothelium of various solid malignancies possibly due to tumor-associated angiogenic factors and endothelial cell sprouting [1,2].

Numerous solid tumor entities including renal cell carcinoma, thyroid cancer, or Hepatocellular Carcinoma (HCC) showed an increased uptake on 18F-PSMA PET/CT [3,4,5]. Therefore, it appears that increased PSMA expression is often associated with poorer prognosis [6]. In pancreatic cancer increased PSMA expression is associated with increased tumor aggressiveness in terms of tumor cell proliferation, tumor growth and a reduced overall survival [7]. Some years ago, except for prostate cancer, PSMA avid lesions were described as random findings. The method is now finding its way more and more outside prostate cancer imaging [8]. Further studies are required to understand the added value of PSMA PET/CT in non-prostatic pathologies [9].

## Figures and Tables

**Figure 1:**
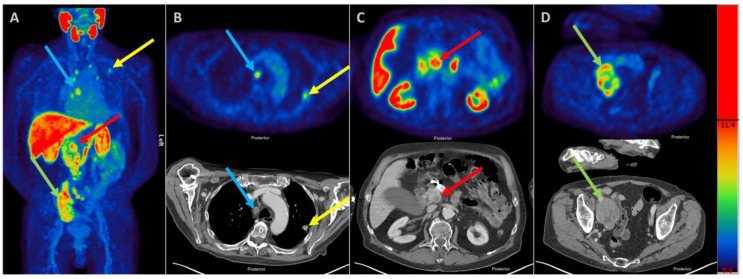
18F-PSMA PET/CT scan of a patient with known prostate cancer (maximum intensity projection (**A**)). Multilocular PSMA positive tumor lesions were revealed in the lung (**B**), the mediastinal (**B**), the pancreas (**C**) as well as in abdominal/iliac lymph nodes (**D**).
